# Intense light-elicited upregulation of miR-21 facilitates glycolysis and cardioprotection through Per2-dependent mechanisms

**DOI:** 10.1371/journal.pone.0176243

**Published:** 2017-04-27

**Authors:** Colleen Marie Bartman, Yoshimasa Oyama, Kelley Brodsky, Ludmila Khailova, Lori Walker, Michael Koeppen, Tobias Eckle

**Affiliations:** 1 Department of Anesthesiology, University of Colorado Denver School of Medicine, Aurora, CO, United States of America; 2 Department of Cell and Developmental Biology, University of Colorado Denver School of Medicine, Aurora, CO, United States of America; 3 Department of Anesthesiology and Intensive Care Medicine, Oita University Faculty of Medicine, Oita, Japan; 4 Division of Cardiology, Department of Medicine, University of Colorado Denver School of Medicine, Aurora, CO, United States of America; 5 Department of Anesthesiology, Ludwig Maximilian University of Munich, Munich, Germany; Indiana University School of Medicine, UNITED STATES

## Abstract

A wide search for ischemic preconditioning (IPC) mechanisms of cardioprotection identified the light elicited circadian rhythm protein Period 2 (Per2) to be cardioprotective. Studies on cardiac metabolism found a key role for light elicited Per2 in mediating metabolic dependence on carbohydrate metabolism. To profile Per2 mediated pathways following IPC of the mouse heart, we performed a genome array and identified 352 abundantly expressed and well-characterized Per2 dependent micro RNAs. One prominent result of our *in silico* analysis for cardiac Per2 dependent micro RNAs revealed a selective role for miR-21 in the regulation of hypoxia and metabolic pathways. Based on this Per2 dependency, we subsequently found a diurnal expression pattern for miR-21 with higher miR-21 expression levels at Zeitgeber time (ZT) 15 compared to ZT3. Gain or loss of function studies for miR-21 using miRNA mimics or miRNA inhibitors and a Seahorse Bioanalyzer uncovered a critical role of miR-21 for cellular glycolysis, glycolytic capacity, and glycolytic reserve. Exposing mice to intense light, a strategy to induce Per2, led to a robust induction of cardiac miR-21 tissue levels and decreased infarct sizes, which was abolished in *miR-21*^*-/-*^ mice. Similarly, first translational studies in humans using intense blue light exposure for 5 days in healthy volunteers resulted in increased plasma miR-21 levels which was associated with increased phosphofructokinase activity, the rate-limiting enzyme in glycolysis. Together, we identified miR-21 as cardioprotective downstream target of Per2 and suggest intense light therapy as a potential strategy to enhance miR-21 activity and subsequent carbohydrate metabolism in humans.

## 1. Introduction

The rotation of the earth and associated light / dark cycles are responsible for entrainment of our circadian system, a dramatic evolutionarily conserved feature affecting uni-cellular organisms to humankind. In the 1970s, researchers began investigating the circadian system in *Drosophila melanogaster*, which led to the identification of gene loci involved in the cellular ‘clock’ such as Period (Per), an important player in the circadian system [1]. In the early 1980’s a multicenter analysis of the limitations of infarct size reported circadian periodicity for acute myocardial infarction (MI), with a peak incidence at 9 AM compared to 9 PM [2]. Since then, the spike in morning MI incidence–after a long period without daylight–has been confirmed repeatedly [3]. These studies suggest that daylight and light-elicited circadian mechanisms play an important role in MI etiology. In agreement with this postulation, there is a well-documented increase of MIs during the darker winter months [4]. Because the onset of MI has a distinct circadian pattern, it has been suggested that disruption of circadian rhythms may contribute to cardiovascular disease [5].

A hallmark of the mammalian circadian pacemaker is its ability to be entrained (i.e., synchronized) by light [6]. Photic stimuli enter the retina and travel via the retinohypothalamic tract to the suprachiasmatic nucleus (SCN) in the brain, where the signals are transduced to the molecular clockwork [7, 8]. Blue wavelengths of light are detected specifically by melanopsin receptors in retinal ganglion cells that leads to the transcriptional induction of Per2 in the SCN and concomitant entrainment. Peripheral tissues display oscillations in Per2 expression similar to those of the brain [9, 10] and thought to be secreted through neurohormonal signaling molecules [7, 11, 12]. Only light with an intensity >180 LUX is able to synchronize the human circadian system [13], whereas intense light (>10,000 LUX) is most effective. In fact, our recent studies found that intense light exposure of mice significantly increased cardiac Per2 levels which was associated with reduced troponin I levels and smaller infarct sizes in an *in-situ* model for myocardial ischemia when compared to room light conditions [10]. Studies in *Per2*^*-/-*^ mice showed a lack of lactate production during myocardial ischemia and the inability to induce glycolytic pathways, a necessary adaptive mechanism during cardiac ischemia [14–16]. When mice were exposed to intense light, the heart had transcriptional induction of glycolytic enzymes from wildtype mice but not *Per2*^*-/-*^ [10]. These findings implicate intense light elicited cardiac Per2 stabilization in endogenous cardioprotection by enhancing oxygen efficient glycolysis and thereby rendering the heart more readily available to withstand ischemia.

Targeting oxygen efficient pathways could be an adaptable strategy for preventing or reducing reperfusion injury during myocardial ischemia in humans. Thus, understanding the interconnection between micro RNAs, circadian rhythmicity, and cellular metabolism during myocardial ischemia has the potential to identify new therapeutic strategies of cardioprotection. While a single micro RNA may target multiple transcripts within a cell type, the contribution of circadian micro RNAs to heart ischemia or metabolism are mostly unknown. To identify micro RNA-based endogenous cardioprotective pathways during MI, we performed a screening experiment to study transcriptional changes of Per2 dependent micro RNAs during cardioprotective ischemic preconditioning (IPC) of the heart. Out of 352 most abundantly expressed micro RNAs, we identified miR-21 amongst the top Per2 dependent micro RNAs that may play a role in metabolic and IPC mediated cardioprotection. In fact, computational analysis revealed a selective role for miR-21 in cardiac ischemia reperfusion injury, hypoxia [17, 18], and metabolic [19, 20] pathways. miR-21 is located on chromosome 17 and is highly conserved in many species, including human, rat, mouse, fish and frog. Remarkably and in line with our findings, miR-21 is one of the most robustly up-regulated miRNAs in hearts after IPC [21]. Moreover, IPC-mediated cardiac protection against ischemia/reperfusion injury was inhibited by knockdown of cardiac miR-21 [22]. Using *in vitro*, murine *in vivo* and human studies, our data suggest miR-21 is a novel downstream target of light and IPC elicited Per2 regulation of cardioprotection and carbohydrate metabolism.

## 2. Methods

### 2.1 Mouse experiments

Experimental protocols were approved by the Institutional Review Board (Institutional Animal Care and Use Committee [IACUC]) at the University of Colorado Denver, USA. They were in accordance with the NIH guidelines for use of live animals. Before experiments, mice were housed for at least 4 weeks in a 14/10-h light-dark cycle to synchronize (entrain) the circadian clock of WT mice to the ambient light-dark cycle. We conducted all mouse experiments at the same time points (ZT 3, ZT15). To eliminate gender- and age-related variations, we routinely used 12- to 16-week-old male mice [10, 23].

### 2.2 Per2^-/-^ mice

*Per2*^*-/-*^ or *miR-21*^*-/-*^ and controls (C57BL/6J or B6129SF1/J) were obtained from the Jackson Laboratories [24, 25]. Characterization and validation were performed as described previously. Homozygous mutant mice are morphologically indistinguishable from their wild-type littermates and both males and females are fertile [10, 23, 25].

### 2.3 Murine model for cardiac ischemic preconditioning [10, 23, 26–32]

Anesthesia was induced (70 mg/kg body weight i.p.) and maintained (10 mg/kg/h) with sodium pentobarbital. Mice were placed on a temperature-controlled heated table (RT, Effenberg, Munich, Germany) with a rectal thermometer probe attached to a thermal feedback controller to maintain body temperature at 37°C. The tracheal tube was connected to a mechanical ventilator (Servo 900C, Siemens, Germany) with pediatric tubing and the animals were ventilated with a pressure controlled ventilation mode (peak inspiratory pressure of 10 mbar, frequency 110 breaths/min, positive end-expiratory pressure of 3 mbar, FiO_2_ = 0.3). Blood gas analysis revealed normal paO_2_ (115±15 mmHg) and paCO_2_ (38±6 mmHg) levels with our ventilator regime. After induction of anesthesia, animals were monitored with a surface electrocardiogram (ECG, Hewlett Packard, Böblingen, Germany). Fluid replacement was performed with normal saline, 0.2 ml/h i.v. The carotid artery was catheterized for continuous recording of blood pressure with a statham element (WK 280, WKK, Kaltbrunn, Switzerland). Operations were performed under an upright dissecting microscope (Olympus SZX12). Following left anterior thoracotomy, exposure of the heart and dissection of the pericardium, the left coronary artery (LCA) was visually identified and an 8.0 nylon suture (Prolene, Ethicon, Norderstedt, Germany) was placed around the vessel. Atraumatic LCA occlusion for IPC studies was performed using a hanging weight system [26, 33]. Successful LCA occlusion was confirmed by an immediate color change of the vessel from light red to dark violet, and of the myocardium supplied by the vessel from bright red to white, as well as the immediate occurrence of ST-elevations in the ECG. During reperfusion, the changes of color immediately disappeared when the hanging weights were lifted and the LCA was perfused again [27–29, 31].

### 2.4 MicroRNA PCR array

Ischemic preconditioning (4 cycles of 5 min ischemia and 5 min reperfusion) with a final reperfusion time of 120 minutes was performed in C57BL/6J (The Jackson Laboratory) or *Per2*^*-/-*^ mice. Heart tissue was snap-frozen with clamps pre-cooled to the temperature of liquid nitrogen. Micro RNA was isolated with Trizol (Invitrogen) and purified using RT^2^ qPCR-Grade miRNA Isolation Kit (SABiosciences-Qiagen). cDNA template was generated using RT^2^ miRNA First Strand Kit (SABiosciences-Qiagen). miRNA expression was performed using RT^2^ miRNA PCR Array Mouse miFinder (SABiosciences-Qiagen).

### 2.5 Transcriptional analysis

Total RNA was isolated from human endothelial cells (HMEC-1) or murine heart tissue by Qiazol Reagent (Qiagen) and chloroform extraction in conjunction with the RNeasy Mini Kit (Qiagen), following manufacturer’s instructions (SA-Biosciences, Qiagen). MicroRNAs were isolated by a secondary ethanol precipitation (100%) of eluate from initial lysate centrifugation through the mini column. MicroRNA elution was quantified by Nanodrop 2000 or Qubit fluorometer 3.0. 100 ng of microRNA eluate was used to make cDNA following the miScript RT II Kit manufacturer’s instructions (Qiagen). cDNA was diluted to 1 to 5 ng/uL for determining transcript levels by real-time quantitative PCR (iCycler; Bio-Rad Laboratories Inc.) and following manufacturer’s instructions for miScript SYBR Green PCR Kit (Qiagen) [34]. Primer sets for Mm-miR-21 (miScript Primer Assay, Qiagen, 5’-UAG CUU AUC AGA CUG AUG UUG A), Hs-miR21 (miScript Primer Assay, Qiagen, 5'-UAG CUU AUC AGA CUG AUG UUG A), Hs-RNU6-2_11 (miScript Primer Assay, Qiagen, used control cat. no. MS00033740, functional in human, mouse, rat, dog, rhesus macaque, cow pig, and sheep) were used following manufactures instructions. Primer sets for human or mouse Per2 were QuantiTect Primer Assay, Qiagen, cat. no. QT00011207, QT00198366 or Invitrogen sense 5´-ACC TGC TCA ACC TCC TTC TG-3´, antisense 5´-ACT ACT GCC TGC CCC ACT TT-3´, respectively. Human or mouse Actb were QuantiTect Primer Assay, Qiagen, cat. no. QT01136772 or Invitrogen sense 5'-CTA GGC ACC AGG GTG TGA T -3', antisense 5'-TGC CAG ATC TTC TCC ATG TC-3'. cDNA from mRNA was generated using iScript (Bio-Rad) and transcript levels were determined by real-time RT-PCR (iCycler; Bio-Rad Laboratories Inc.) [34]. The PCR reactions contained 1 μM sense and 1 μM antisense oligonucleotides with SYBR Green (Bio-Rad, 170–8880). Each target sequence was amplified using increasing numbers of cycles of 94°C for 1 min, 58°C for 0.5 min, 72°C for 1 min. Quantification of transcript levels was determined by real-time RT-PCR (iCycler; Bio-Rad Laboratories Inc.).

### 2.6 Light exposure in mice

Mice were exposed to intense light (10,000 LUX, Lightbox simulating day light, Uplift Technologies DL930 Day-Light 10,000 Lux SAD, full spectrum) for 3 h [10] or one week and compared to mice maintained at room light [200 LUX [10]]. Mice were housed in a 14/10-h light-dark cycle to synchronize (entrain) the circadian clock of WT mice to the ambient light-dark cycle. We conducted all mouse experiments at same time points (ZT 3, ZT15).

### 2.7 Isolation of fibroblasts

Heart tissue from C57BL6/J mice was minced and digested using Collagenase Type II solution (Worthington Biochemical Corporation) at 37°C, 100 rpm, collecting the supernatant every 10 minutes for 90 minutes and replacing with fresh collagenase solution until heart tissue fully digested. Fibroblasts were isolated after plating and incubation of the cell suspension in a cell culture incubator with 5% CO2 for 2 h. 2 h upon plating alive and healthy fibroblasts were adhered to the dish. After cells reached confluency, cells were exposed to normoxia (21% oxygen) or hypoxia (1% oxygen using preequilibrated media for 6 h [35]) and immediately resuspended in Trizol for miRNA analysis.

### 2.8 Isolation of adult cardiomyocytes [10, 32]

8–12 weeks old C57BL6/J mice were anesthetized and the heart was quickly removed from the chest cavity and immediately placed in ice-cold KHB buffer. After weighing, the aorta was cannulated and the heart were perfused with Ca^2+^-free KHB for 3 min followed by 8–12 min perfusion with Ca^2+^-free KHB containing collagenase. After perfusion, ventricles were removed, minced and incubated with the collagenase solution for an additional 3–7 min. The cells were filtered through a nylon mesh (60 μm) and collected in a 15-ml sterile tube. Myocytes were washed and calcium was slowly re-introduced in a stepwise fashion. Finally, cells were resuspended in MEM supplemented with FBS, BDM, penicillin, and ATP and plated on laminin. After healthy myocyte adhesion to laminin-coated plates, media was exchanged for serum-free MEM and cells were exposed to normoxia (21% oxygen) or hypoxia (1% oxygen using preequilibrated media for 1 h [36]) the next day and immediately resuspended in Trizol for miRNA analysis [10].

### 2.9 Endothelial cells

C57BL/6 mouse primary cardiac endothelial cells were obtained from Cell Biologics (C57-6024) and handled following manufacturer’s instructions in complete mouse endothelial cell medium supplemented with VEGF, ECGS, heparin, EGF, hydrocortisone, L-glutamine, antibiotic-antimycotic solution, and FBS (M1168). After cells reached confluency, cells were exposed to normoxia (21% oxygen) or hypoxia (1% oxygen using preequilibrated media for 3 h [37]) and immediately resuspended in Trizol for miRNA analysis.

### 2.10 Metabolic analysis in miR-21 gain or loss of function

For gain of function experiments, we used a MISSION hsa-miR-21 Mimic (Sigma-Aldrich, cat. no. HMI0372). The miR-21 Mimic was delivered to human microvascular endothelial cells (HMEC-1) using DharmaFect I Transfection Reagent (Dharmacon). For loss of function experiments, we used an anti-miR-21 (Qiagen, MIMAT0000076: 5'UAG CUU AUC AGA CUG AUG UUG A, MIMAT0004494: 5'CAA CAC CAG UCG AUG GGC UGU, MIMAT0004494: 5'CAA CAC CAG UCG AUG GGC UGU; MIMAT0000076: 5'UAG CUU AUC AGA CUG AUG UUG A) and a miScript Inhibitor Negative Control (Qiagen, cat. no. 1027271). The anti-miR-21 was delivered to HMEC-1 using HiPerFect Transfection Reagent (Qiagen). Cells were seeded at a density of 30,000 cells/well prior to transfection. Cells were synchronized by serum starvation, followed by glycolytic stress test using a Seahorse Bioanalyzer XF24 per manufacturer’s protocol. Our glycolytic stress test protocol was optimized for use in HMECs using final concentrations of 10 mM glucose, 1.0 uM oligomycin, and 50 mM 2-deoxy-D-glucose in XF Assay Medium (Seahorse Biosciences). The Seahorse Bioanalyzer measures extracellular acidification rates (ECAR) in live HMECs in response to treatment with these compounds.

### 2.11 Light exposure humans

We obtained approval from the Institutional Review Board (Colorado Multiple Institutional Review Board [COMIRB]) for our human studies and prior to these studies we obtained written informed consent from each individual. Data presented were from 8 healthy individuals (5 males, 3 females). The average age was 29.5 years old (range 23–41 yo). All but one individual identified as a caffeine drinker. The average number of hours slept prior to intense light exposure did not differ from the week of intense light exposure (6.4h). Healthy human volunteers were exposed to 30 minutes of intense light (Square One Wake Up Light, NatureBright, Day-Light 10,000 Lux) in the morning at 8:30 AM for 5 consecutive days. A blood draw was performed before light exposure on the first day (8:30 AM) and 5 days after light exposure (9.00 AM). Blood was collected in EDTA-plasma tubes and spun at 3,000 rpm for 8 minutes to separate plasma. Plasma samples were analyzed for miR-21 levels and PFK (phosphofructokinase) activity. Light boxes were a generous gift from Joshua Chen, NatureBright.

### 2.12 Phosphofructokinase (PFK) activity

Phoshpofructokinase activity was measured using a PFK Activity Colorimetric Assay Kit (BioVision, cat. no. K776-100), adhering to manufacturer’s instructions.

### 2.13 Data analysis

Data were compared by Student’s t test. Values are expressed as mean (SD) from 3–6 animals/individual cell experiments or 8 healthy human volunteers per condition. The chosen numbers per group was based on findings in previous studies and a subsequent samples size analysis. The studies are designed to be able to reject the null hypothesis that the population means of the experimental and control groups are equal with probability (power) 0.8. The Type I error probability associated with this test of this null hypothesis is 0.05. *P*<0.05 was considered statistically significant. For all statistical analysis, GraphPad Prism 5.0 software for Windows XP was used. The authors had full access to and take full responsibility for the integrity of the data. All authors have read and agree to the manuscript as written.

## 3. Results

### 3.1 Differential and Per2 dependent regulation of micro RNA miR-21 after ischemic preconditioning (IPC)

**Fig 1:**
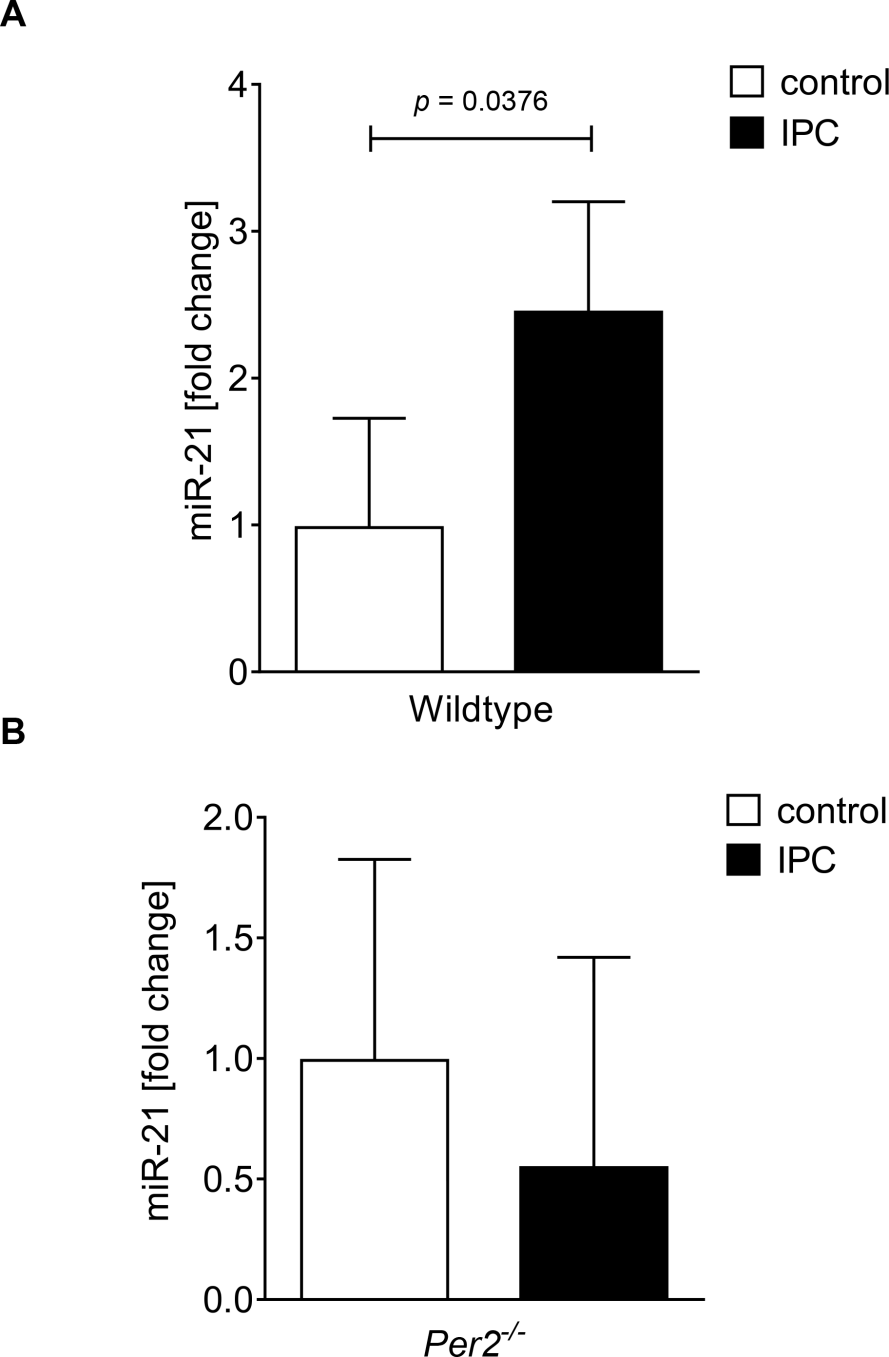
Studies of miR-21 regulation in wiltype and *Per2*^*-/-*^ mice after cardiac ischemic preconditioning. Wildtype (**A**) or *Per2*^*-/-*^ mice (**B**) were exposed to cardiac IPC, consisting of 4 x 5 minutes of ischemia followed by 5 minutes of reperfusion each, followed by a final reperfusion time of 120 min. Heart tissue was snap-frozen with clamps pre-cooled to the temperature of liquid nitrogen. Total RNA was isolated from murine heart tissue using Qiazol Reagent and separated into mRNA and miRNA components following manufactures instructions (SA-Biosciences, Qiagen). cDNA from miRNA was generated using miScript RT II kits (Qiagen) and transcript levels were determined by real-time RT-PCR (iCycler; Bio-Rad Laboratories Inc.; mean±SD, n = 3).

Our previously published studies showed abolished cardioprotection by IPC in *Per2*^*-/-*^ mice [10]. Based on these studies, we pursued a wide micro RNA screen of cardiac Per2 dependent micro RNAs (**Table 1**). Out of 352 micro RNAs analyzed, 186 were regulated in both wildtype and *Per2*^*-/-*^ mice, 65 were only regulated in wildtype and 22 were only regulated in *Per2*^*-/-*^ mice. As shown in Table 1, differential regulation of putative Per2 dependent micro RNAs revealed almost exclusively cardioprotective pathways. Ingenuity analysis revealed a selective role for miR-21 in protection from reperfusion injury of the heart. After identification of miR-21 as a potential downstream target of Per2 mediated cardioprotection, we confirmed a Per2 dependent miR-21 regulation in cardiac tissue from wildtype or *Per2*^*-/-*^ mice; while IPC resulted in a 2.4-fold induction of miR-21 in wildtype mice (**Fig 1A**), no upregulation was observed in *Per2*^*-/-*^ mice (**Fig 1B**). *Taken together*, *these data demonstrate that IPC induced Per2 regulates cardiac miR-21*.

**Table 1:** Per2 dependent micro RNAs during cardiac ischemic preconditioning (IPC). Shown are the 65 differentially regulated and Per2 dependent micro RNAs identified after IPC treatment of wildtype and *Per2*^*-/-*^ mice.

IP regulated	FC	FC	function	publication	species
mmu-miR-16	4.0278	-1.2311	prevents cardiac hypertrophy	J Cell Mol Med. 2015 Mar;19(3):608–19	R
mmu-miR-409-5p	6.9644	1.9053	*role in the heart not studied yet*	** **	** **
mmu-miR-154	5.6962	1.4241	protect against cardiac dysfunction	Sci Rep. 2016 Mar 1;6:22442	M
mmu-miR-326	5.6569	1.9053	*role in the heart not studied yet*		
mmu-miR-24	5.579	1.7291	inhibits cardiomyocyte apoptosis	J Cell Mol Med. 2015 Jan;19(1):103–12	M
mmu-miR-27b	5.2054	1.6702	increases angiogenesis heart	Vase Cell. 2015 Jun 24;7:6	M
mmu-miR-146a	5.1337	1.9725	inhibits cardiomyocyte apoptosis	Mol Ther Nucleic Acids. 2016 Mar 15;5:e296	M
mmu-miR-126-3p	4.7568	-1.1329	*role in the heart not studied yet*		
mmu-miR-25	4.6268	1.2397	protects cardiomyocytes	lntJ Mol Sc i. 2015 Mar 10;16(3):5420–33.	R
mmu-miR-23b	4.4383	1.7654	upregulated in heart failure	Eur J Heart Fail. 2016 Apr 12.	H
mmu-miR-186	4.4383	-1.021	diagnosis of unstable angina pectoris	Eur Heart J. 2014 Aug 14;35(31):2106–14	H
mmu-miR-191	4.4076	1.3195	biomarkers for Ml	Biomed Res Int. 2014;2014:418628	H
mmu-miR-150	4.2871	1.3195	protect against cardiac fibrosis	Cell Physiol Biochem. 2016 May 17;38(6):2103–2122	M
mmu-miR-342-3p	4.2281	1.4948	biomarker heart failure	Eur J Heart Fail. 2013 Oct;15(10):1138–47	H
mmu-miR-28	4.1411	1.0867	*increases cardiomyocyte apoptosis*	Eur Rev Med Pharmacal Sci. 2015; 19 (5)	M
mmu-miR-99a	4.1411	1.1487	prevents cardiac hypertrophy	PloS One. 2016 Feb 25;11(2):e0148480	M
mmu-miR-322	4.1125	-1.0867	protects against cardiac dysfunction	Biochim Biophys Acta. 2016 Apr;1862(4):611–21	M
mmu-miR-30a	4.084	1.7053	protect against cardiac dysfurnction	Mol Cell Biochem. 2013 Jul;379(1–2):1–6	M
mmu-miR-23a	4.084	1.9185	*increases cardiac hypertrophy*	J Bioi Chem. 2012 Jan 2;287(1):589–99	M
mmu-miR-181b	4.0278	1.9053	upregulated in heart failure	Eur J Heart Fail. 2016 Apr 12. doi	H
mmu-miR-101a	3.9724	1.1567	protects cardiac fibroblasts	lntJ Biochem Cell Bioi. 2015 Aug;65: 155–64	R
mmu-miR-505	3.9313	1.1368	regenerative neonatal mouse heart	Cell Biochem Biophys. 2014 Sep;70(1):635–42	M
mmu-miR-20a	3.9177	-1.3755	prevents cardiac hypertrophy	PloS One. 2013 Nov13;8(11):e79133	R
mmu-miR-199b	3.8637	-1.3755	*increases cardiac hypertrophy*	Cardiovasc Res. 2016 May 15;110(2):258–67	M
mmu-miR-324-5p	3.8106	1.5157	attenuates cardiomyocyte apoptosis	Cell Death Dis. 2015 Dec 3;6:e2007	M
mmu-miR-30c	3.7842	1.6818	prevents cardiac hypertrophy	Circ Res. 2009 Jan 30;104(2):170–8	M
mmu-miR-208b	3.7064	-2.4116	biomarkers for LV remodling	lntJ Mol Sc i. 2014 Apr 4;15(4):5774–88. doi	H
mmu-miR-301a	3.6808	-1.5583	*role in the heart not studied yet*		
mmu-let-7d	3.6301	1.3287	*role in the heart not studied yet*		
mmu-miR-582-5p	3.6175	-4.4229	*role in the heart not studied yet*		
mmu-miR-466d-3p	3.5554	-1.1173	*role in the heart not studied yet*		
mmu-miR-155	3.5308	-1.0425	*increases cardiac hypertrophy*	J Am Heart Assoc. 2016 Apr 22;5(4)	M
mmu-miR-532-3p	3.4224	1.9931	*increases cardiomyocyte apoptosis*	Cell Death Dis. 2015 Mar 12;6:e1677	M
mmu-miR-30e	3.4105	-1.0644	*role in the heart not studied yet*		
mmu-miR-126-5p	3.3636	-1.0425	*role in the heart not studied yet*		
mmu-miR-350	3.3636	1.1647	*increases cardiac hypertrophy*	Biochim Biophys Acta. 2013 Jan;1832(1)	R
mmu-miR-148b	3.3404	1.3566	*role in the heart not studied yet*		
mmu-miR-541	3.3173	1.3947	prevents cardiac hypertrophy	Cell Death Dis. 2014 Apr 10;5:e1171	M
mmu-miR-301b	3.3173	-1.1647	*role in the heart not studied yet*		
mmu-miR-181d	3.2944	1.0425	*role in the heart not studied yet*		
mmu-miR-106b	3.2266	-1.2924	*role in the heart not studied yet*		
mmu-miR-151-5p	3.2266	1.0353	prevents arrythm ias in Ml	Pl oS One. 2013 Sep 9;8(9):e72985	R
mmu-miR-128a	3.2043	1.6133	*role in the heart not studied yet*		
mmu-miR-425	3.1821	1.4044	regu lates ANP product ion	Mol Cell Bioi. 2016 May 16. pii: MCB.01114-15	H
mmu-miR-152	3.1821	1	*role in the heart not studied yet*		
mmu-miR-99b	3.1383	1.7901	*role in the heart not studied yet*		
mmu-miR-22	3.1167	1.4845	Cardioprotective	Gene. 2016 Mar 15;579(1):17–22	R
mmu-miR-467c	3.1167	-1.2924	*role in the heart not studied yet*		
**mmu-miR-21**	**3.0738**	**-1.5369**	**IP heart, cardioprotection**	Cardiovasc Res. 2010 Aug 1;87(3):431–9	M
mmu-miR-872	3.0738	-1.0867	*role in the heart not studied yet*		
mmu-let-7i	3.0738	1.021	*role in the heart not studied yet*		
mmu-miR-18a	3.0738	-1.5692	*role in the heart not studied yet*		
mmu-let-7c	3.0525	1.4743	*role in the heart not studied yet*		
mmu-miR-17	3.0525	-1.257	*role in the heart not studied yet*		
mmu-let-7f	3.0314	-1.366	*role in the heart not studied yet*		
mmu-miR-467e	3.0105	-1.6818	*role in the heart not studied yet*		
mmu-miR-219	2.2974	-4.1411	*role in the heart not studied yet*		
mmu-miR-675-5p	1.9521	3.1932	*role in the heart not studied yet*		
mmu-miR-302c	1.7654	24.4201	*role in the heart not studied yet*		
mmu-miR-742	1.0281	3.9449	*role in the heart not studied yet*		
mmu-miR-216a	-1.057	4.4076	*role in the heart not studied yet*		
mmu-miR-343	-3.1711	-1.244	*role in the heart not studied yet*		
mmu-miR-744	-3.3636	-1.014	*role in the heart not studied yet*		
mmu-miR-679	-3.4943	-1.4191	*role in the heart not studied yet*		
mmu-miR-292-3p	-5.8159	-1.4743	*role in the heart not studied yet*		

IP = Ischemic Preconditioning, FC = Fold Change.

### 3.2 Diurnal expression pattern of miR-21 in the murine heart and lung

**Fig 2:**
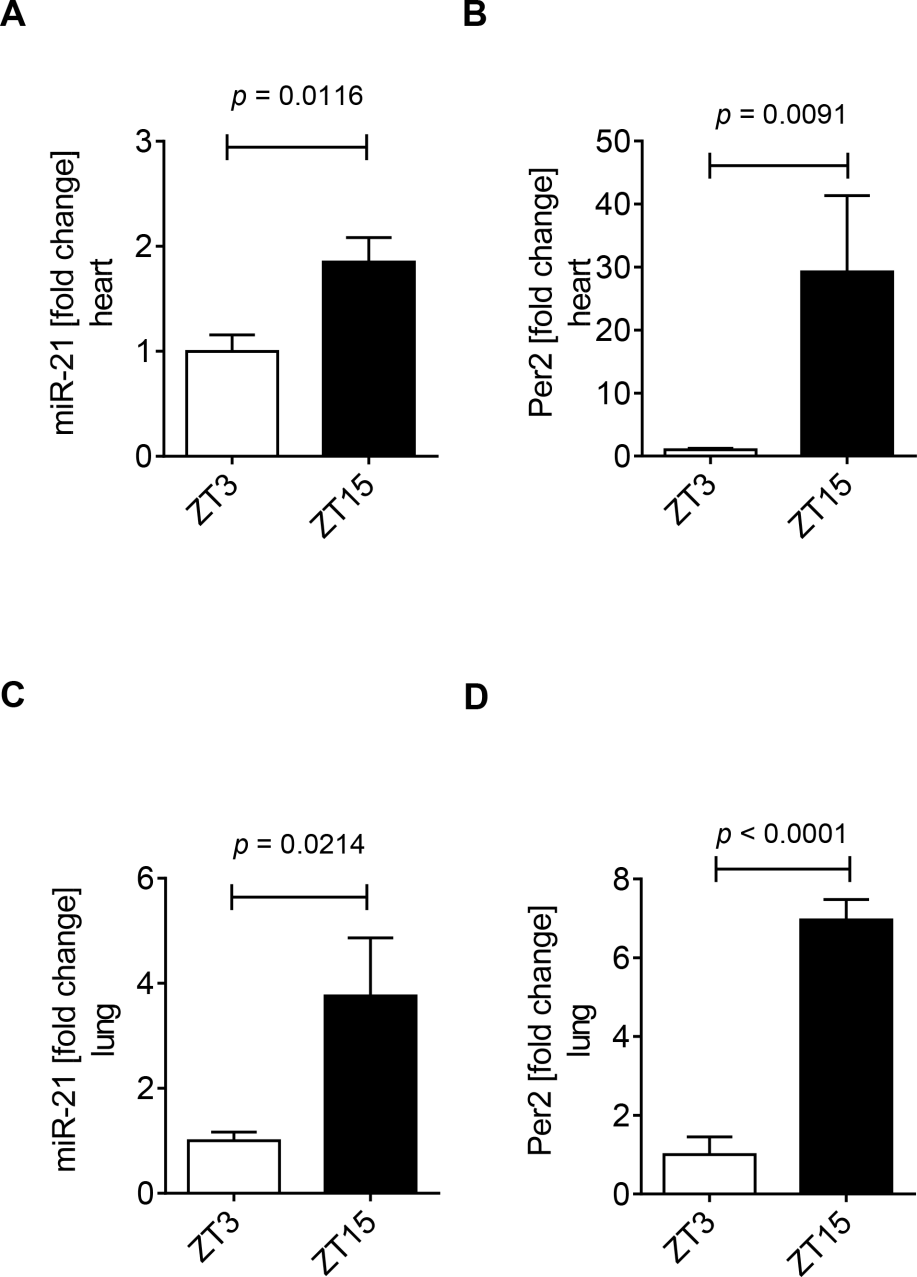
Diurnal expression of miR-21 in murine hearts and lungs. Analysis of cardiac (**A**) or lung (**B**) mir-21 and Per2 levels from wildtype mice at Zeitgeber Time (ZT) 3 or ZT15. Total RNA was isolated from murine heart or lung tissue using Qiazol Reagent and separated into mRNA and miRNA components following manufactures instructions (SA-Biosciences, Qiagen). cDNA from miRNA was generated using miScript RT II kits (Qiagen) and transcript levels were determined by quantitative real-time RT-PCR (iCycler; Bio-Rad Laboratories Inc.; mean±SD, n = 3, p<0.05).

Considering cardiac miR-21 is regulated in a Per2 dependent manner, and cardiac Per2 has a diurnal oscillation pattern, we next investigated the expression pattern of this micro RNA over a 12h period. Hearts from wildtype mice were harvested at Zeitgeber time (ZT) 3 or ZT15. We found significantly higher cardiac miR-21 expression levels at ZT15 compared to ZT3 (1.8-fold increase from ZT3 to ZT15, **Fig 2A**). Consistent with our previously published studies [10], analysis of the same heart tissue confirmed significantly higher cardiac Per2 mRNA levels at ZT15 compared to ZT3 (29-fold increase from ZT3 to ZT15, **Fig 2B**). To verify a diurnal nature of miR-21 we analyzed another organ in addition to the heart. Indeed, analysis of lung tissue from these wildtype mice at the indicated times revealed lung miR-21 and Per2 mRNA levels significantly higher at ZT15 than ZT3, which was consistent with our findings in the heart (lung miR-21 3.7-fold increase and lung Per2 mRNA 6.9-fold increase from ZT3 to ZT15, respectively, **Fig 2C and 2D**). *Taken together*, *our studies show that murine miR-21 expression oscillates over the circadian day in heart and lungs (ZT3 vs ZT15*, *p < 0*.*05)*, *like Per2 which implies a putative circadian expression pattern of miR-21*.

### 3.3 miR-21 is exclusively upregulated in hypoxic cardiac endothelial cells

**Fig 3:**
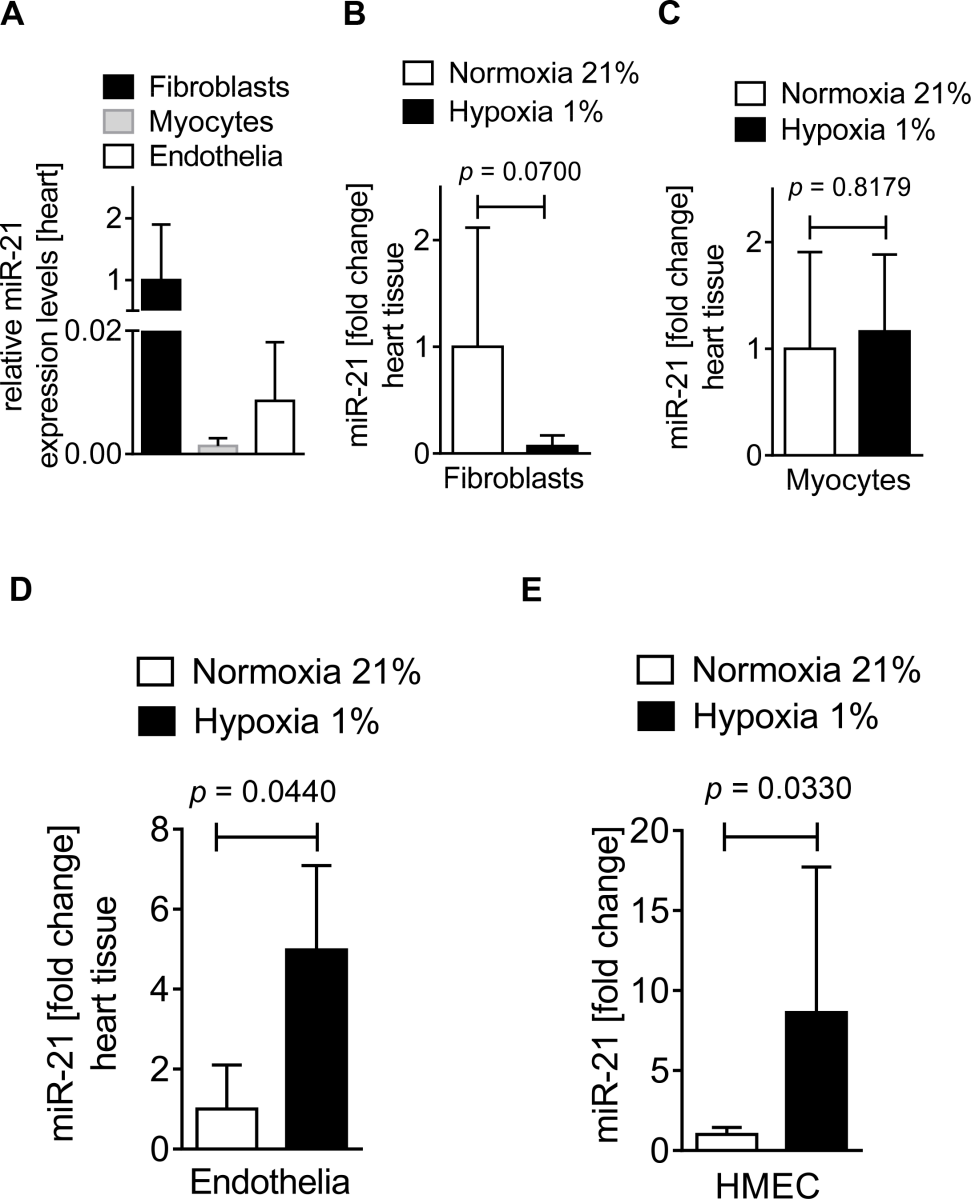
miR-21 expression in different cardiac tissues at baseline and during hypoxia. Fibroblasts or myocytes were isolated from C57BL6/J mouse hearts and endothelial cells isolated from C57/BL6 mice were purchased from Cell Biologics for analyzing miR-21 expression at baseline or hypoxic (1% oxygen) conditions. miRNA was isolated using RNeasy Mini Kit (Qiagen), cDNA was generated using miScript RT II kits (Qiagen), and transcript levels were determined by quantitative real-time RT-PCR (iCycler; Bio-Rad Laboratories Inc.). (**A**) Relative miR-21 expression levels in C57BL6/J mouse isolated cardiac fibroblasts, myocytes, and endothelia at baseline (mean±SD, n = 3, *not significant*). (**B**) miR-21 expression in cardiac fibroblasts subjected to normoxia or hypoxia for 6 h (mean±SD, n = 6, *not significant*). (**C**) miR-21 expression in cardiac myocytes subjected to normoxia or hypoxia for 1 h (mean±SD, n = 3, *not significant*). (**D**) miR-21 expression in cardiac endothelia subjected to normoxia or hypoxia for 6 h (mean±SD, n = 6, p<0.05). (**E**) miR-21 expression in human endothelia (HMEC-1) subjected to normoxia or hypoxia for 6 h (mean±SD, n = 6, p<0.05).

After confirming that miR-21 is a Per2 regulated micro RNA with a diurnal expression pattern in heart and lungs, we next investigated which cardiac cell type expressed miR-21 during conditions of low oxygen availability. Based on previous findings that miR-21 is predominantly expressed in cardiac fibroblasts [38], we obtained fibroblasts, myocytes or endothelial cells from wildtype mouse hearts. In fact, analysis of relative miR-21 expression levels indicated an abundant expression of miR-21 in cardiac fibroblasts when compared to other cardiac cell types (**Fig 3A**). However, since fibroblasts play a dominant role during remodeling [39] but not during the acute phase of myocardial ischemia and reperfusion [32], we next exposed isolated murine cardiac fibroblasts, myocytes or endothelial cells to 1% oxygen (hypoxia). As shown in **Fig 3B and 3C**, no significant regulation of miR-21 was found in fibroblasts or myocytes upon hypoxia exposure when compared to cells at ambient oxygen levels (normoxia). However, isolated murine cardiac endothelial cells exposed to hypoxia revealed a robust and significant upregulation of miR-21 (4.9-fold increase compared to ambient oxygen levels, **Fig 3D**). Further analysis using human microvascular endothelial cells (HMEC-1) confirmed a miR-21 upregulation in hypoxia (8.6-fold increase in 1% hypoxia, **Fig 3E**). *Taken together*, *while miR-21 is predominantly expressed in cardiac fibroblasts at baseline*, *only cardiac endothelial cells revealed a significant upregulation of miR-21 upon hypoxia exposure*. *These data suggest that endothelial expressed miR-21 plays a critical role during conditions of low oxygen availability*, *such as myocardial ischemia*.

### 3.4 miR-21 is critical for cellular glycolysis, glycolytic capacity, and glycolytic reserve

**Fig 4:**
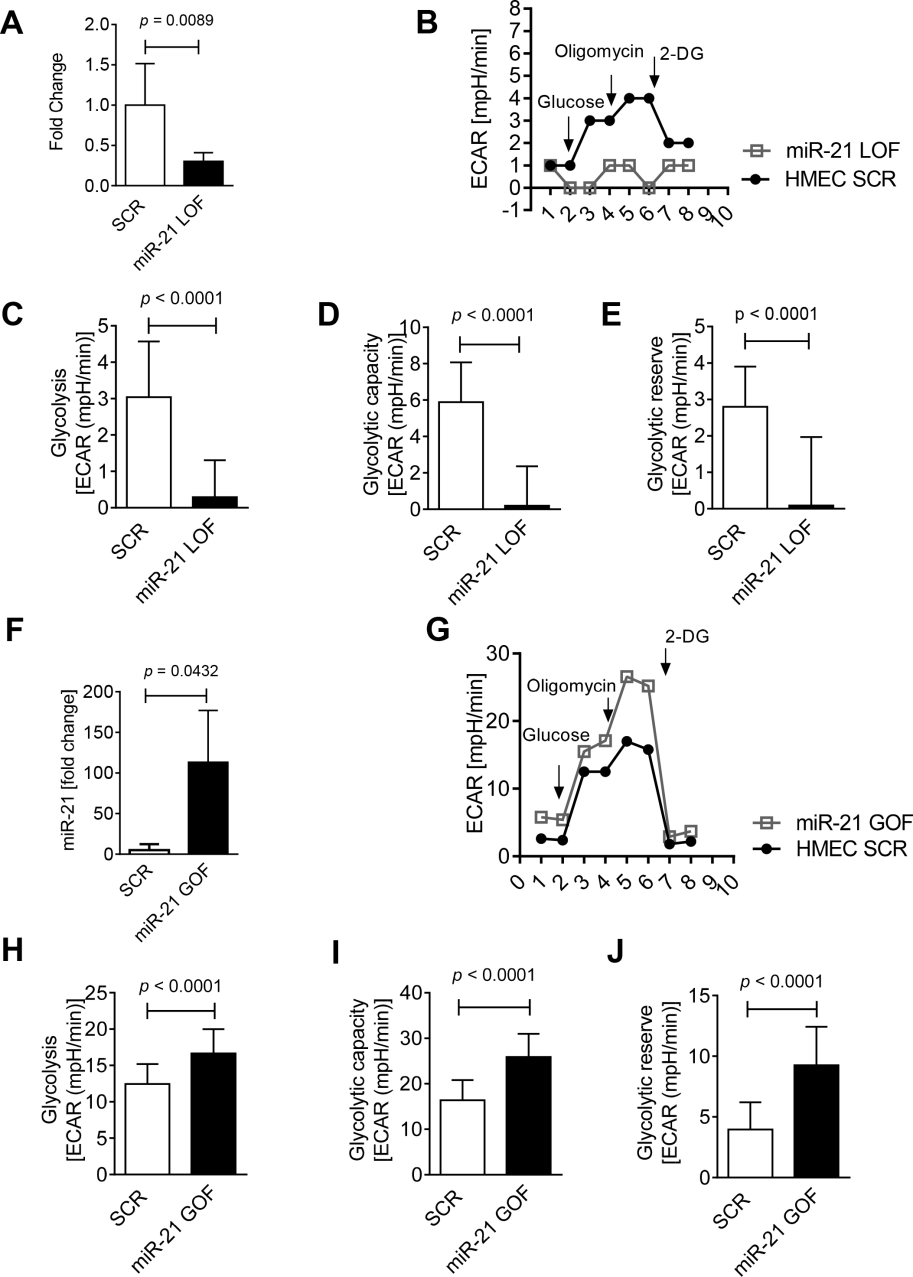
Glycolysis in miR-21 gain or loss of function human endothelial cells. **(A)** Knockdown confirmation in anti-mir-21 (loss of function, LOF) treated human endothelial cells (HMEC-1). (**B-E**) Glucose metabolism from control (miScript Inhibitor Neg. Control, scrambled [SCR]) and anti-mir-21 (LOF) treated HMEC-1. (**F**) Overexpression in miR-21Mimic (gain of function [GOF]) treated HMEC-1. (**G-J**) Glucose metabolism from control (miScript miRNA Mimic Neg. Control, SCR) and miR-21Mimic (GOF) treated human endothelial cells (HMEC-1). Cells were seeded at a density of 100,000 cells/well. Glycolysis assay was performed using glycolysis stress test kit from Seahorse Biosciences according to manufacturer’s protocol using the XF24 instrument. The extracellular acidification rate (ECAR) response to glucose, oligomycin and 2-DG was measured (mean±SD, n = 6, p<0.05).

After confirming that miR-21 is a hypoxia regulated micro RNA with predominant upregulation in hypoxic cardiac endothelial cells, we next analyzed the role of miR-21 in known Per2 regulated pathways. Our recent studies found an important role of light elicited Per2 in controlling glycolysis during myocardial ischemia [10, 12, 23]. To understand a potential role of miR-21 in glycolysis, we first performed loss of function (LOF) studies using miR-21 inhibitors. Anti-miR-21 inhibitors were transfected into human microvascular endothelial cells (HMEC-1), a cell line well characterized for hypoxic, metabolic and Per2 pathways [10]. We first confirmed a knockdown of miR-21 in HMEC-1s and found a 70% reduction of miR-21 expression (**Fig 4A**). In miR-21 knockdown HMEC-1s, we assessed glycolysis, glycolytic capacity, and glycolytic reserve using a glycolytic stress test and Seahorse Bioanalyzer (**Fig 4B**). Loss of miR-21 significantly reduced glycolysis (10.7-fold, **Fig 4C**), glycolytic capacity (31-fold, **Fig 4D**) and glycolytic reserve (31-fold, **Fig 4E**). In contrast, our gain of function (GOF) studies done by overexpressing a miR-21 mimic (22-fold overexpression, **Fig 4F**) significantly increased glycolysis (1.3-fold, **Fig 4G and 4H**), glycolytic capacity (1.6-fold, **Fig 4I**) and glycolytic reserve (2.3-fold, **Fig 4J**) in HMEC-1s. *Taken together*, *these studies demonstrate that miR-21 is necessary to maintain glycolysis*, *critical for the cell to maximally respond to glycolytic demand (glycolytic capacity)*, *and pertinent for glucose reserves to be available for use through glycolysis beyond baseline (glycolytic reserve)*.

### 3.5 *miR-21*^*-/-*^ mice have larger infarct sizes in myocardial ischemia and reperfusion

**Fig 5:**
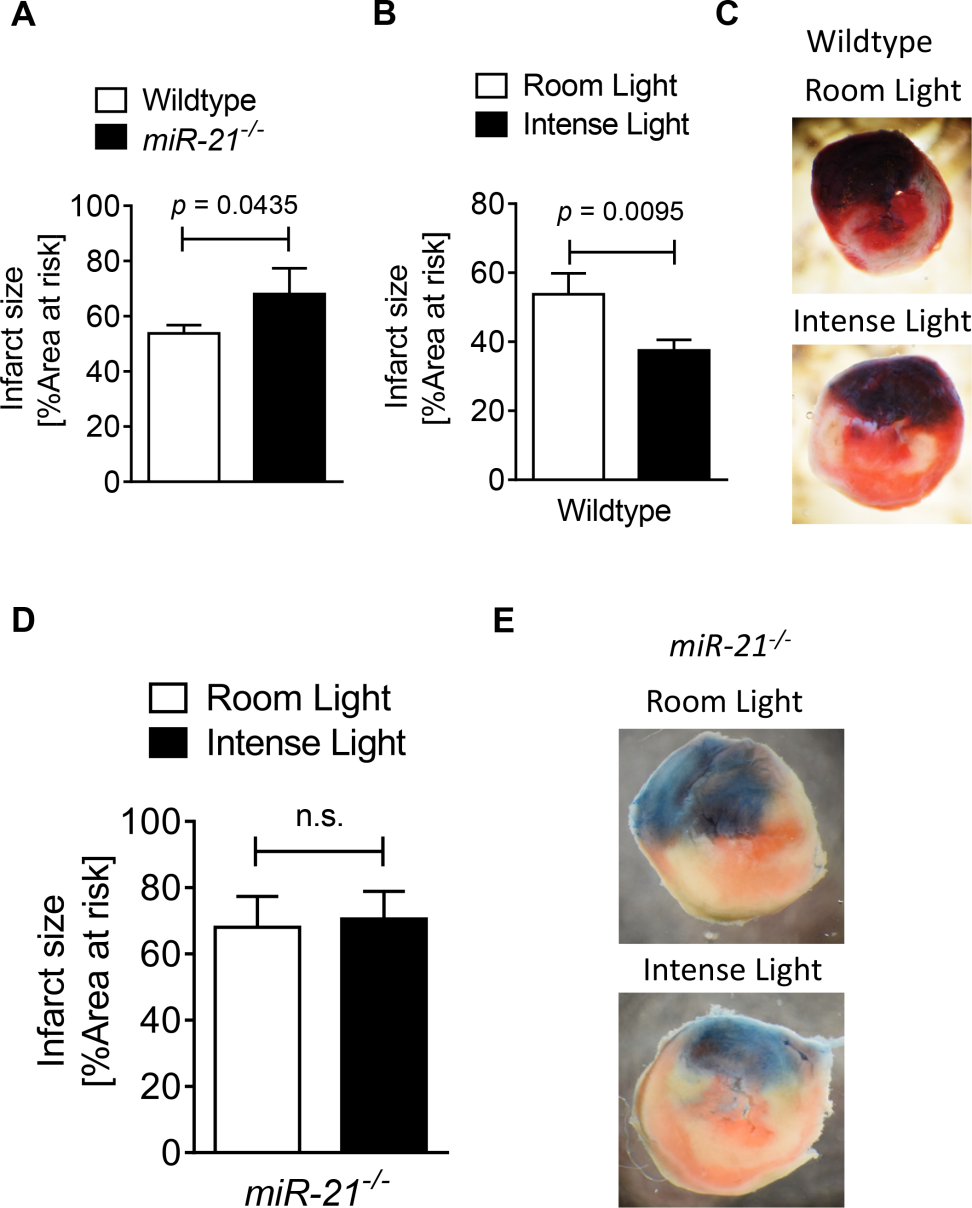
Light elicited cardioprotection in wildtype and *miR-21*^*-/-*^ mice. **(A-E)** Mice underwent 60 min of ischemia and 120 min of reperfusion at room light (200LUX) or after exposure to 3 hours of intense light (10,000 LUX). Infarct sizes were measured by double staining with Evan’s blue and triphenyl-tetrazolium chloride. Infarct sizes are expressed as the percent of the area at risk (AAR) that underwent infarction. (**A**) Infarct sizes in wildtype or *miR-21*^*-/-*^ mice at room light conditions (mean±SD, n = 4, p<0.05). (**B, C**) Infarct sizes in wildtype mice after exposure to intense light for 3 h compared to room light conditions. (mean±SD, n = 4, p<0.05). (**C**) Representative infarct staining in hearts from wildtype mice exposed to intense light or room light prior to *in situ* myocardial ischemia and reperfusion (blue, retrograde Evan’s blue staining; red and white, area at risk; white, infarcted tissue). (**D, E**) Infarct sizes in *miR-21*^*-/-*^ mice exposed to intense light or room light prior to *in situ* myocardial ischemia followed by reperfusion (mean±SD, n = 4, *not significant*). (**E**) Representative infarct staining in hearts from *miR-21*^*-/-*^ mice exposed to intense light or room light prior to *in situ* myocardial ischemia reperfusion (blue, retrograde Evan’s blue staining; red and white, area at risk; white, infarcted tissue).

After confirming that miR-21 was necessary for Per2 regulated pathways such as glycolysis, we next investigated the role of miR-21 in myocardial ischemia and reperfusion injury. Thus, we first exposed *miR-21*^*-/-*^ or control mice (B6129SF1/J) to myocardial ischemia and reperfusion injury. As shown in **Fig 5A**, *miR-21*^*-/-*^ mice had significant larger infarct sizes after 60 minutes of ischemia and 120 min of reperfusion than their littermate controls (*miR-21*^*-/-*^: 68 ± 9% vs. B6129SF1/J: 53.75 ± 6%). *Taken together*, *these studies show that miR-21 is functional and cardioprotective in myocardial ischemia and reperfusion injury*.

### 3.6 Light elicited cardioprotection is abolished in *miR-21*^*-/-*^ mice

After confirming a cardio-protective role of miR-21 in myocardial ischemia and reperfusion injury, we next investigated miR-21 as a potential downstream target of Per2 in myocardial ischemia and reperfusion injury. Previous studies found light exposure to increase cardiac Per2 and mimic IPC mediated cardioprotection in a Per2 dependent manner [10]. Based on our findings that IPC increased cardiac miR-21 in a Per2 dependent manner, we next exposed wildtype controls or *miR-21*^*-/-*^ mice to 3 h of intense light prior to myocardial ischemia and reperfusion injury as done previously in *Per2*^*-/-*^ mice [10]. As shown in **Fig 5B and 5C**, light exposure significantly reduced infarct sizes in wildtype controls (Intense light vs. room light: 37.5 ± 6.1% vs. 53.75 ± 6%). However, identical intense light exposure conditions in *miR-21*^*-/-*^ mice failed to induce cardioprotection (Room light vs intense light: 68 ± 9% vs. 70.5 ± 8.3%, **Fig 5D and 5E**). *Taken together*, *these data show that intense light mediated cardioprotection is abolished in miR-21*^*-/-*^ *mice and suggest that miR-21 is a downstream target of light elicited Per2 in cardioprotection from myocardial ischemia and reperfusion injury*.

### 3.7 Intense light exposure induces cardiac miR-21

**Fig 6:**
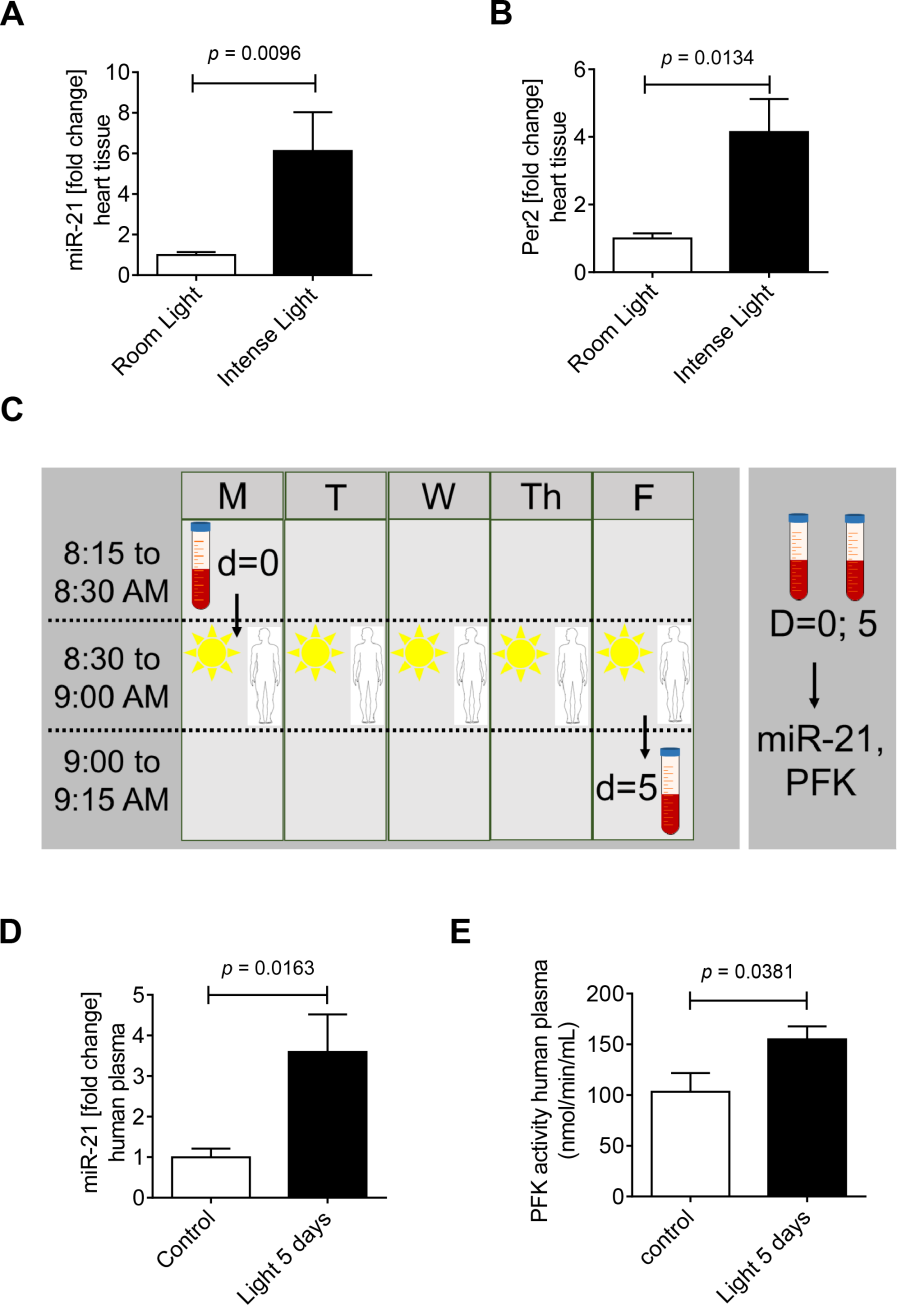
Effects of intense light on miR-21 regulation in mice and human subjects. **(A-C)** Wildtype mice were exposed to broad spectrum intense light (10,000 lux) for 7 days (LD 14:10) and compared to controls that were maintained at room light (200 lux, LD 14:10). Total RNA was isolated from murine hearts using Qiazol Reagent and separated into mRNA and miRNA components following manufactures instructions (SA-Biosciences, Qiagen). cDNA from miRNA was generated using miScript RT II kits (Qiagen) and miR-21 or Per2 transcript levels were determined by real-time RT-PCR (iCycler; Bio-Rad Laboratories Inc.; mean±SD, n = 3, p<0.05). (**D-F**) 7 Healthy human volunteers were exposed to 30 minutes of intense blue light (Square One Wake Up Light, NatureBright, Day-Light 10,000 Lux) in the morning at 8:30 AM on 5 consecutive days. A blood draw was performed before light exposure on the first day (8:30 AM) and 5 days after light exposure (9.00 AM). Plasma samples were analyzed for miR-21 levels and PFK (phosphofructokinase) activity (mean±SD, n = 7, p<0.05).

After finding that Per2 dependent miR-21 was critical for Per2 regulated glycolysis or light elicited cardioprotection, we next extended our *in vivo* studies to determine if exposing mice to intense light–a strategy to overexpress cardiac Per2 [10]–induces cardiac miR-21. To test this, wildtype mice were exposed to one week of intense light at 10,000 lux (14h light/10h dark, **Fig 6A**) and compared to room light at 200 lux (14h light/10h dark). Housing mice for one week at intense light significantly increased cardiac miR-21 (6.1-fold, **Fig 6B**) when compared to room light housing. As a control for light treatment we also analyzed Per2 levels and found a robust and significant induction of cardiac Per2 mRNA levels (4.1-fold, **Fig 6C**), as observed in earlier studies on cardiac Per2 protein [10]. *Taken together*, *exposing mice to intense light increases miR-21 levels in cardiac tissues*, *like Per2 mRNA*, *which supports that miR-21 could be indeed a circadian micro RNA downstream of Per2*.

### 3.8 Intense light exposure increases miR-21 and PFK activity in healthy human volunteers

After we found intense light regulation of cardiac miR-21, we next pursued studies on light therapy in healthy human volunteers. In fact, earlier studies found increased Per2 levels in buccal swaps from human volunteers upon light treatment [40]. Thus, we exposed eight healthy volunteers (3 females, 5 males) to 30 min of intense light therapy from 8:30 until 9:00 AM for 5 days (**Fig 6C**). Blood was drawn on day one at 8:30 AM before any intense light exposure and on day 5 at 9:00 AM after intense light exposure. Plasma samples were used to isolate micro RNAs and to determine miR-21 plasma levels. Five days of intense light therapy significantly increased miR-21 plasma levels in human subjects (3.5-fold, **Fig 6D**). Based on findings that miR-21 overexpression was associated with increased glycolysis *in vitro*, we next determined plasma phosphofructokinase activity, the key regulatory enzyme in the glycolytic pathway. Here, intense light exposure led to a 49% increase of PFK activity (**Fig 6E**). *Taken together*, *one week of intense light exposure in human subjects increases miR-21 levels in blood plasma samples which is associated with increased phosphofructokinase activity*.

## 4. Discussion

**Fig 7:**
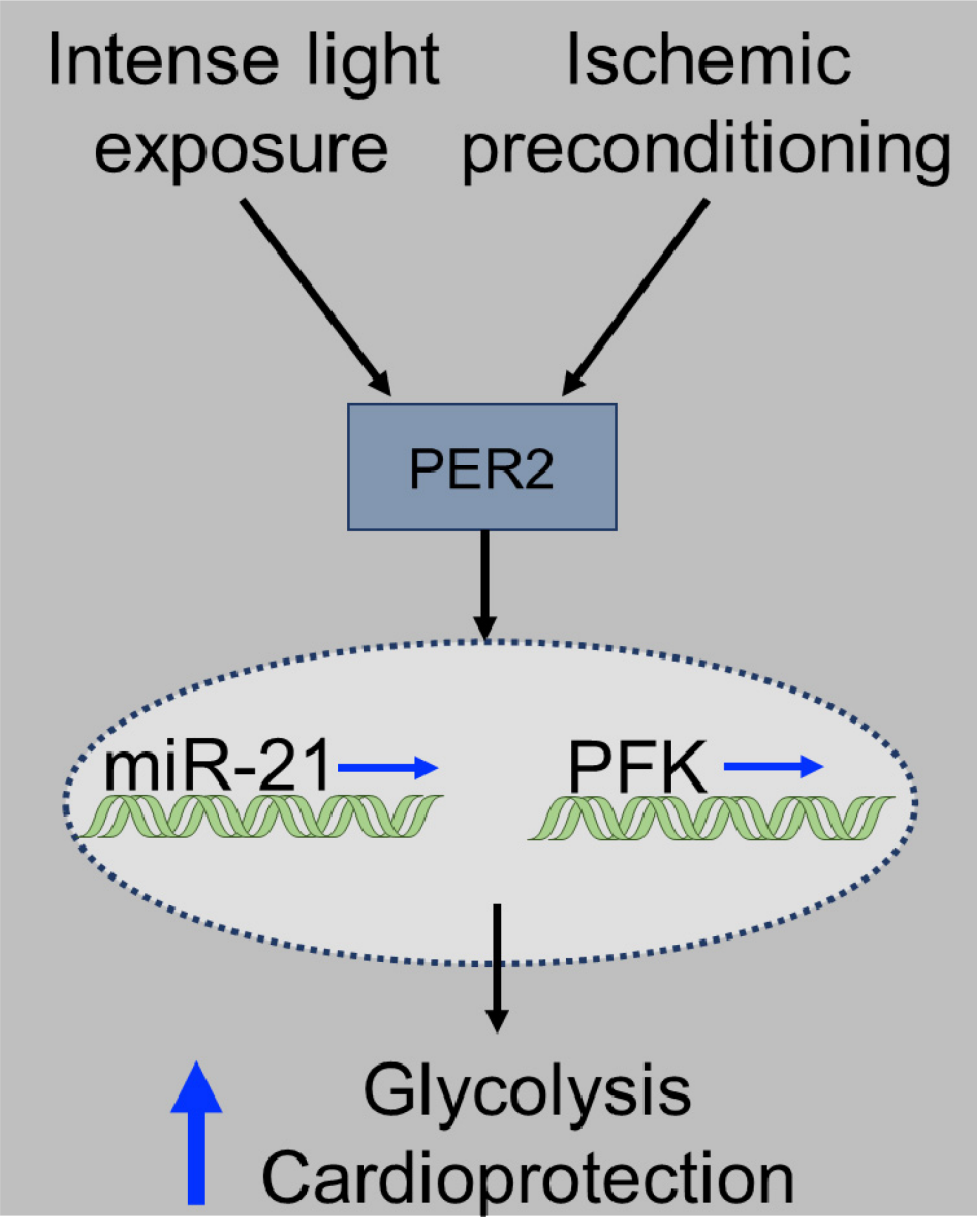
Proposed model of IPC or light induced miR-21 and glycolysis. Both IPC and light can induce Per2 in human or murine tissues. In a Per2 dependent manner miR-21 and phosphofructokinase (PFK) are transcriptionally induced which finally leads to increased PFK activity, glycolytic capacity and cardioprotection.

In the present study, we pursued identification of microRNAs that could mimic circadian rhythm protein Period (Per2) mediated cardioprotection. Profiling the Per2 dependent expression of 352 micro RNAs following cardioprotective ischemic preconditioning (IPC) of the heart indicated an exclusive role for miR-21. Analysis of three cardiac tissues revealed hypoxia induced miR-21 predominantly in cardiac endothelial cells. Studies on miR-21 expression revealed a Per2 dependent and putative circadian profile. Using miR-21 LOF or GOF in HMEC-1s revealed a critical role of miR-21 for Per2 regulated cellular glycolysis. Studies on myocardial ischemia and reperfusion injury revealed larger infarct sizes and abolished light elicited Per2 cardioprotection in *miR-21*^*-/-*^ mice. Intense light exposure in mice or humans increased miR-21 levels and light exposure in humans also increased phosphofructokinase activity. Taken together, these studies suggest manipulation of miR-21 through intense light or IPC to increase glycolysis, a potential therapeutic strategy for myocardial ischemia (**Fig 7)**.

While miR-21 is one micro RNA that has a strong involvement in cardioprotective pathways such as IPC [22] or metabolic pathways [19], other identified microRNAs could be possible candidates for Per2 mediated pathways. As such studies on miR-22 (**Table 1**) found that exosomes, enriched with miR-22, were secreted by mesenchymal stem cells following cardiac IPC and mobilized to cardiomyocytes where they reduced their apoptosis due to ischemia. In addition, while miR-21 was found to play a key role in cardiac IPC [22, 41] or preventing apoptosis in cardiomyocytes [18], long-term elevation of miR-21 may be also be detrimental to the organ by promoting the development of fibrosis in an acute cardiac allograft transplantation model [42]. Interestingly, these findings would support the idea that an intact circadian pattern of circadian proteins with high peaks but also low troughs would be most beneficial. In fact, studies on sepsis outcomes in rats during constant light or constant darkness conditions found both conditions to be detrimental [43]. Therefore, light exposure probably needs to be adapted to the time-of-day where intense light late at night could be more detrimental than beneficial. In fact, clinical studies in humans have found light at night to disrupt circadian rhythms and to negatively affect metabolism [44, 45].

Research on circadian microRNAs in the heart has been extremely limited. We propose miR-21 to be circadian based on a Per2 dependent regulation and findings on a diurnal expression pattern like that of Per2. However, high temporal resolution gene expression analysis would be necessary to further support that miR-21 is indeed circadian [46]. In general, studies on circadian micro RNAs are scarce. However, a recent elegant study on sepsis which also has been shown to be time of day dependent, discovered miR-155 as circadian micro RNA with profound effects on circadian function and circadian induction of cytokines by LPS [47]. If a *miR-21* knockdown in mice could have similar effects on circadian function seems compelling but would need to wait further characterization of period lengths in constant darkness or constant light conditions.

MiR-21 is predominantly expressed in cardiac fibroblasts when compared with other cell types of the heart [39]. Therefore, it is surprising to find a role for miR-21 in cardioprotection from acute myocardial ischemia and reperfusion injury. In fact, cardiac fibroblasts are considered as key therapeutic target in cardiac remodeling [39]. However, during acute myocardial ischemia and reperfusion other cells types, such as inflammatory cells, myocytes or endothelial cells are more important. As such, a recent study on adenosine signaling in IPC of the heart found abolished or dampened cardioprotection by IPC in mice with a tissue specific deletion of the adenosine A2B receptor in cardiomyocytes or endothelia, respectively. Based on these observations we exposed primary fibroblasts, cardiomyocytes or endothelial cells from C57BL6/J mouse hearts to hypoxia and analyzed miR-21 expression. Here we found that miR-21 was exclusively upregulated during conditions of low oxygen availability, indicating that endothelial expressed miR-21 is critical during acute myocardial ischemia. In fact, a recent study on myocardial ischemia and reperfusion injury found protective effects of miR-21 in endothelial injury, further supporting our findings [48].

The critical role of miR-21 in glycolysis seems surprising. However, it was shown that one of Per2 mediated mechanisms is controlling transcription as a cofactor [49]. In fact, the transcription factor hypoxia inducible factor 1 alpha (HIF1A) is the key regulator of glycolysis [50] and studies have shown that Per2 and HIFA are bound together during ischemia of the heart [10]. Furthermore, miR-21 has also been found to be a HIF1A target gene [18]. Data from these studies would therefore suggest that Per2-HIF1A complex is responsible for the transcriptional regulation of miR-21 during myocardial ischemia. In line with these findings, recent studies on miR-21 in small lung cancer cells revealed a similar connection between miR-21, glycolysis and HIF1A [51]. However how miR-21 controls glycolysis would need further mechanistic studies. In addition, while our studies demonstrated more PFK activity in humans exposed to our intense light protocol, the kinetic of elevated PFK and its downstream effects are not known. Thus, further studies would need to be done in humans, such as *in vivo* labeled tracers, to determine if elevated PFK activity does indeed increase glycolytic flux and glycolytic reliance. If done, these studies could also help elucidate the length of time PFK activity is elevated and functional after light exposure.

In our studies, using miR-21 deficient mice, we found significantly increased larger infarct sizes when compared to controls. In contrast, other studies on miR-21 null mice did not find any significant differences in infarct sizes during myocardial ischemia and reperfusion injury [52, 53]. While several differences in methodologies might have contributed to the contrary findings, the most prominent difference was the ischemia time. In our studies mice were exposed to 60 minutes of ischemia, while the reported paper used 30 minutes of ischemia. Indeed, others have found marked differences in cardioprotective mechanisms using different ischemia times [54]. Despite these contrary findings, other studies have shown a protective role for miR-21 in ischemic preconditioning [55], postconditioning [41], or protection form ischemia and reperfusion injury of the heart [56], using miR-21 inhibitors or mimetics, supporting our current findings.

Light exposure has been found to increase Per2 and glycolytic enzymes and decrease infarct size and troponin levels during MI in mice [10]. As such, intense light induction of Per2 regulated cardiac miR-21 is not very surprising. However, intense light therapy in the regulation of microRNAs has not been described yet. While studies on cardioprotective effects of light exposure in humans are missing, light induced cardioprotective miR-21 could be one mechanism by which intense light exposure reduced myocardial damage in murine studies [10]. In fact, we found intense light elicited cardioprotection to be abolished in *miR-21*^*-/-*^ mice. Similarly, earlier studies found abolished light elicited cardioprotection in *Per2*^*-/-*^ mice [10]. Together, these findings support our hypothesis, that miR-21 is downstream of Per2 and indicate a critical role for miR-21 in light or Per2 mediated cardioprotection. Therefore, thinking of light as potential therapy could represent a novel strategy in the treatment of myocardial ischemia by modulation of cardioprotective micro RNAs.

The light exposure system we used in the present human studies was a light box emitting intense (10,000 LUX) and blue light, since light (melanopsin) receptors are most sensitive to blue light and therefore most effective in synchronizing circadian rhythms [57]. However, we did not investigate whether the intensity, the blue light specifically, or both, determined these changes in miR-21 or PFK. Further studies would be needed to identify which component is necessary for the therapeutic potential of intense light therapy. Regardless, studies in humans on seasonal affective disorder found low intense blue light as effective as standard bright light (10 000 LUX, [58]).

To our knowledge nobody has analyzed human metabolic changes upon intense light therapy yet. The effects of intense light therapy in humans are recognized and already widely used. As such intense light therapy is used to treat winter depression [59, 60], but also might have effects on preventing delirium [61] or might improve sleep in general [62, 63]. Our findings show that intense light significantly increased miR-21 in human plasma samples which was associated with increased phosphofructokinase activity, the key enzyme of glycolysis. These findings indicate that our *in vitro* and murine *in vivo* findings are translatable into a human system. More detailed studies on intense light therapy in humans will hopefully help us to further dissect those mechanisms. However, it needs to be pointed out that it is unclear if light activated glycolysis in humans would be indeed cardioprotective as seen in murine studies [10].

Taken together, using a wide microRNA screen in *Per2*^*-/-*^ mice we found cardioprotective miR-21 to be Per2 dependent. Like Per2, miR-21 was light inducible, mediated light elicited cardioprotecion and regulated glycolysis in human endothelial cells or in human subjects. Intense light exposure could therefore present a novel and promising approach to activate cardioprotective pathways in humans.
